# Supplementary material for: Comparative Transcriptome Analyses of Different Rheum officinale Tissues Reveal Differentially Expressed Genes Associated with Anthraquinone, Catechin, and Gallic Acid Biosynthesis
Source: Genes (Basel). 2022 Sep 5;13(9):1592. doi: 10.3390/genes13091592 (PMC9498579; doi:10.3390/genes13091592)
Supplement: Supplementary file 1 [file genes-13-01592-s001.zip › Table S3.pdf]

Table S3. DEGs involved in secondary metabolic pathways in KEGG enrichment analysis.

NO.	Secondary metabolic pathway	ko_ID	DEGs Number		
			R *vs.* L	R *vs.* S	S *vs.* L
1	Phenylpropanoid biosynthesis	ko00940	11	19	15
2	Terpenoid backbone biosynthesis	ko00900	5	5	-
3	Flavonoid biosynthesis	ko00941	5	6	12
4	Carotenoid biosynthesis	ko00906	5	4	4
5	Isoquinoline alkaloid biosynthesis	ko00950	2	2	2
6	Tropane, piperidine and pyridine alkaloid biosynthesis	ko00960	3	1	-
7	Stilbenoid, diarylheptanoid and gingerol biosynthesis	ko00945	1	4	4
8	Sesquiterpenoid and triterpenoid biosynthesis	ko00909	1	-	1
9	Zeatin biosynthesis	ko00908	1	3	3
10	Diterpenoid biosynthesis	ko00904	-	1	-
11	Brassinosteroid biosynthesis	ko00905	2	1	2
12	Flavone and flavonol biosynthesis	ko00944	1	-	2
13	Ubiquinone and other terpenoid-quinone biosynthesis	ko00130	3	3	3

Note: R, S, and L represent roots, stems and leaves, respectively.
