# Supplementary material for: Comparative Transcriptome Analyses of Different Rheum officinale Tissues Reveal Differentially Expressed Genes Associated with Anthraquinone, Catechin, and Gallic Acid Biosynthesis
Source: Genes (Basel). 2022 Sep 5;13(9):1592. doi: 10.3390/genes13091592 (PMC9498579; doi:10.3390/genes13091592)
Supplement: Supplementary file 1 [file genes-13-01592-s001.zip › Table S5.pdf]

Table S5. The candidate genes involved in anthraquinone biosynthesis of *R. officinale.*

Pathway	Enzyme Name	Enzyme Symbol	EC	KO number	Number of Genes
Shikimate pathway	3-Deoxy-7-phosphoheptulonate synthase	*DAHPS*	EC:2.5.1.54	K01626	10
	3-Dehydroquinate synthase	*DHQS*	EC:4.2.3.4	K01735	3
	3-dehydroquinate dehydratase / shikimate dehydrogenase	*DHQD/SDH*	EC:4.2.1.10 1.1.1.25	K13832	11
	Shikimate kinase	*SK*	EC:2.7.1.71	K00891	2
	3-Phosphoshikimate 1-carboxyvinyltransferase	*EPSPs*	EC:2.5.1.19	K00800	7
	Chorismate synthase	*CS*	EC:4.2.3.5	K01736	12
	Isochorismate synthase	*ICS*	EC:5.4.4.2	K02552	1
	O-succinylbenzoate synthase	*MenC*	EC:5.4.4.2 2.2.1.9 4.2.99.20 4.2.1.113	K14759	1
	o-succinylbenzoate---CoA ligase	*MenE*	EC:6.2.1.26	K14760	1
	1,4-Dihydroxy-2-naphthoyl-CoA synthase	*MenB*	EC:4.1.3.36	K01661	2
	1,4-Dihydroxy-2-naphthoyl-CoA hydrolase	*MenI*	EC 3.1.2.28	K19222	0
MEP pathway	1-Deoxy-D-xylulose-5-phosphate synthase	*DXS*	EC:2.2.1.7	K01662	6
	1-Deoxy-D-xylulose-5-phosphate reductoisomerase	*DXR*	EC:1.1.1.267	K00099	6
	2-C-Methyl-D-erythritol 4-phosphate cytidylyltransferase	*CMS*	EC:2.7.7.60	K00991	1
	4-(cytidine 5'-diphospho)-2-C-methyl-D-erythritol kinase	*CMK*	EC:2.7.1.148	K00919	1
	2-C-Methyl-D-erythritol 2,4-cyclodiphosphate Synthase	*MCS*	EC:4.6.1.12	K01770	0
	(E)-4-Hydroxy-3-methylbut-2-enyl-diphosphate synthase	*HDS*	EC:1.17.7.1 1.17.7.3	K03526	8
	4-hydroxy-3-methylbut-2-en-1-yl diphosphate reductase	*HDR*	EC:1.17.7.4	K03527	3
MVA pathway	Acetyl-CoA C-acetyltransferase	*AACT*	EC:2.3.1.9	K00626	28
	Hydroxymethylglutaryl-CoA synthase	*HMGS*	EC:2.3.3.10	K01641	22
	Hydroxymethylglutaryl-CoA reductase (NADPH)	*HMGR*	EC:1.1.1.34	K00021	22
	Mevalonate kinase	*MVK*	EC:2.7.1.36	K00869	0
	Phosphomevalonate kinase	*PMK*	EC:2.7.4.2	K00938	4
	Diphosphomevalonate decarboxylase	*MVD*	EC:4.1.1.33	K01597	11
	Isopentenyl-diphosphate Delta-isomerase	*IDI*	EC:5.3.3.2	K01823	6
Polyketide pathway	Type III polyketide synthase	*PKS III*	-	-	7
Glycosylation	UDP-glucosyl transferase	*UGT*	EC:2.4.1.-	K13496	2
CYPs	Cytochrome P450	-	-	-	107
	NADPH-cytochrome P450 reductase	-	EC:1.14.14.37	-	5
