# Supplementary material for: Comparative Transcriptome Analyses of Different Rheum officinale Tissues Reveal Differentially Expressed Genes Associated with Anthraquinone, Catechin, and Gallic Acid Biosynthesis
Source: Genes (Basel). 2022 Sep 5;13(9):1592. doi: 10.3390/genes13091592 (PMC9498579; doi:10.3390/genes13091592)
Supplement: Supplementary file 1 [file genes-13-01592-s001.zip › Table S7.pdf]

Table S7. The repeat number of SSR repeat types.

Repeat type	Repeat number			
	**2~5**	**6~10**	**11~15**	**> 15**
Dinucleotide	0	3868	268	169
Trinucleotide	16742	1822	53	18
Tetranucleotide	7641	55	0	0
Pentanucleotide	2220	11	2	0
Hexanucleotide	94704	33	6	0
Total	121307	5789	329	187
Percentage (%)	95.06	4.54	0.26	0.15
