# Supplementary figures and images for: Comparative Transcriptome Analyses of Different Rheum officinale Tissues Reveal Differentially Expressed Genes Associated with Anthraquinone, Catechin, and Gallic Acid Biosynthesis
Source: Genes (Basel). 2022 Sep 5;13(9):1592. doi: 10.3390/genes13091592 (PMC9498579; doi:10.3390/genes13091592)

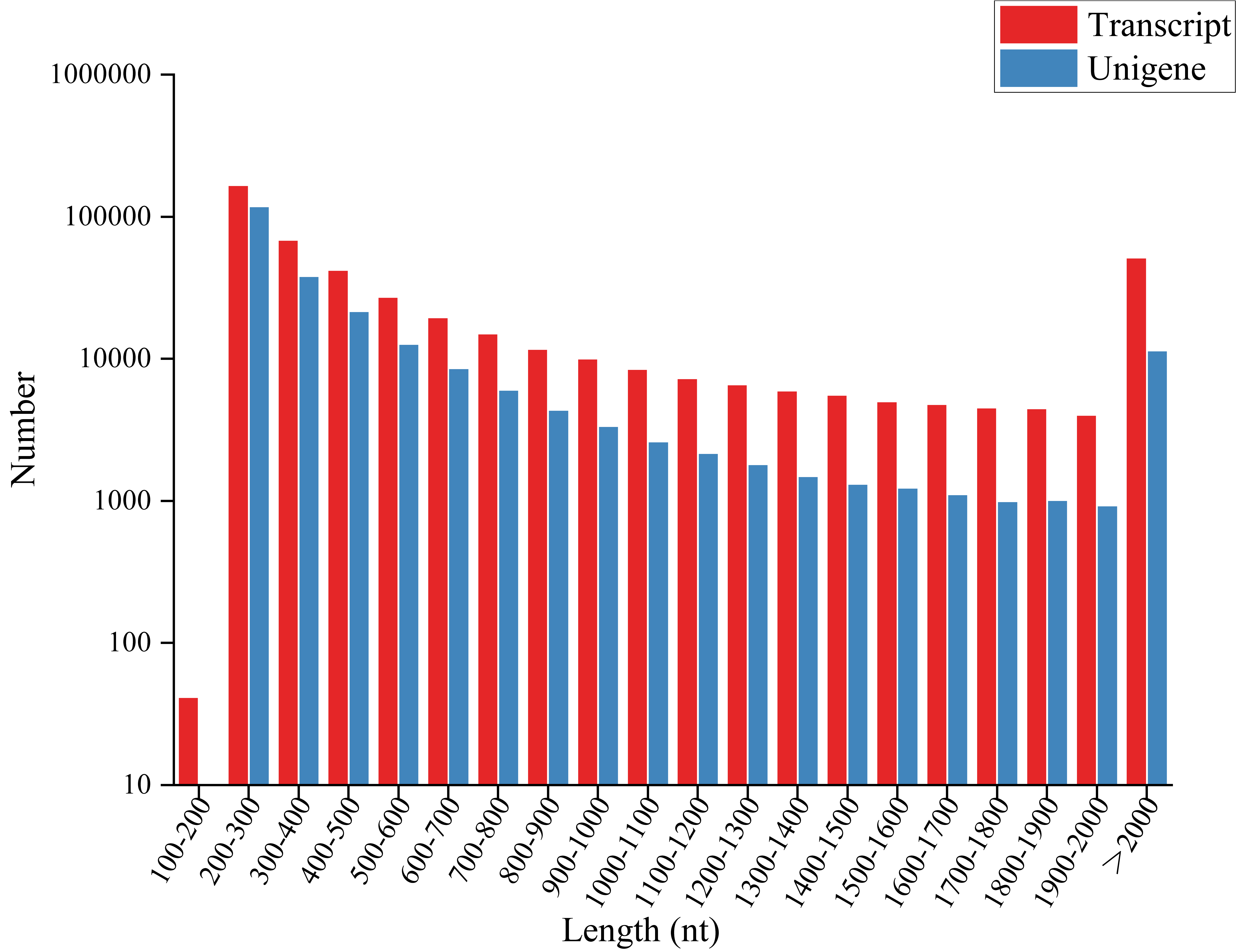

Supplement: Supplementary file 1 [file genes-13-01592-s001.zip › Figure S1.tif]

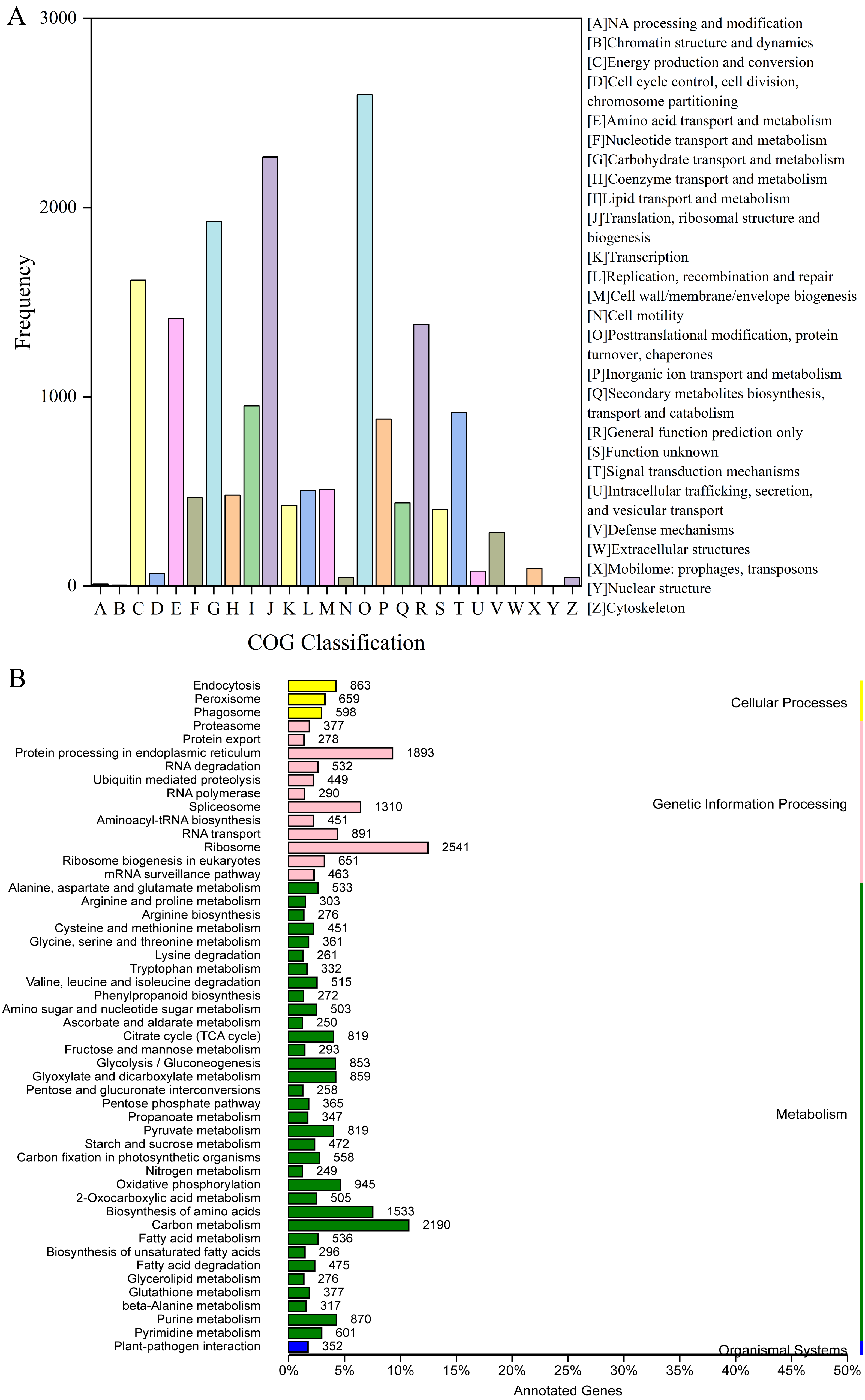

Supplement: Supplementary file 1 [file genes-13-01592-s001.zip › Figure S2.tif]

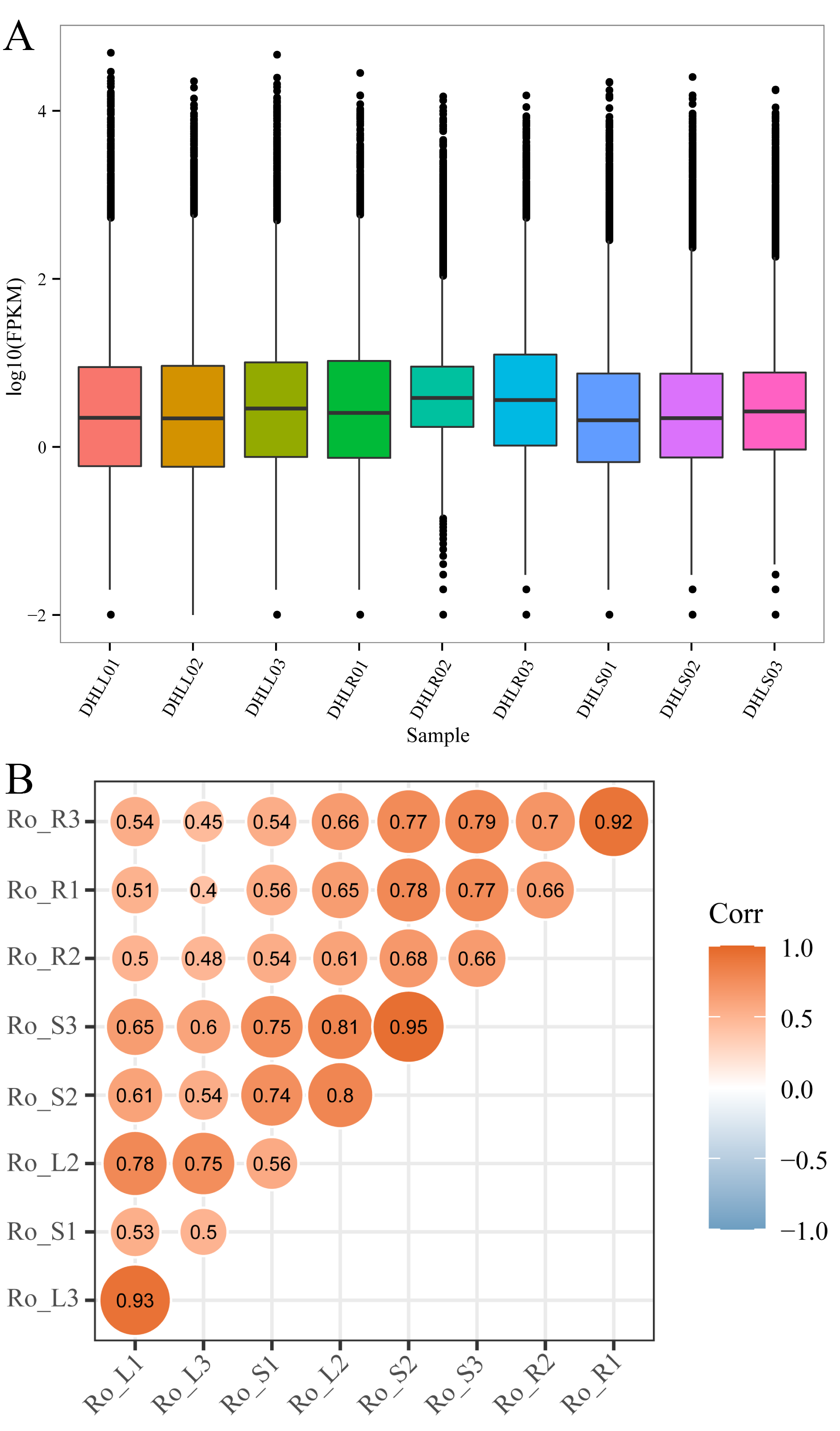

Supplement: Supplementary file 1 [file genes-13-01592-s001.zip › Figure S3.tif]

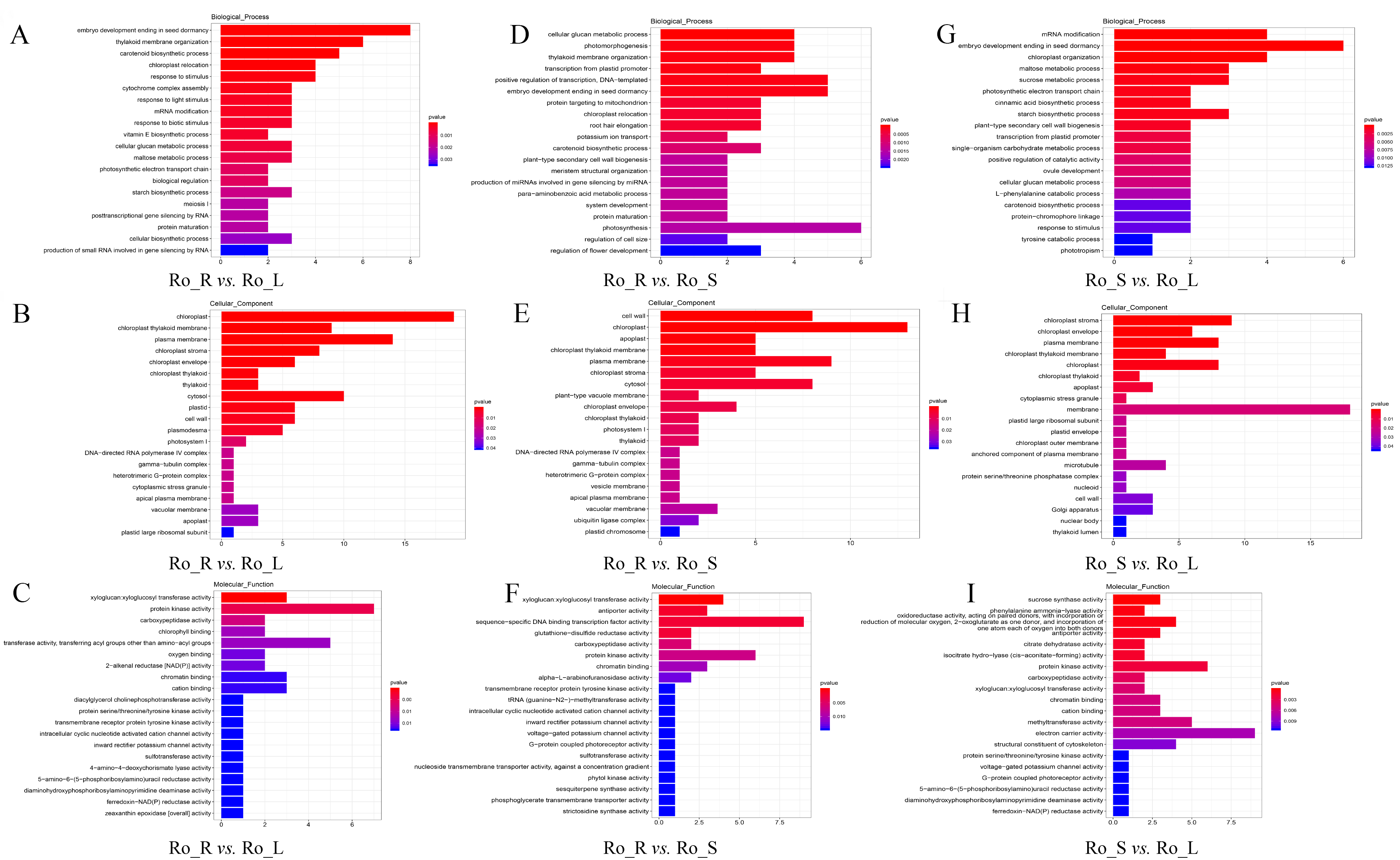

Supplement: Supplementary file 1 [file genes-13-01592-s001.zip › Figure S4.tif]

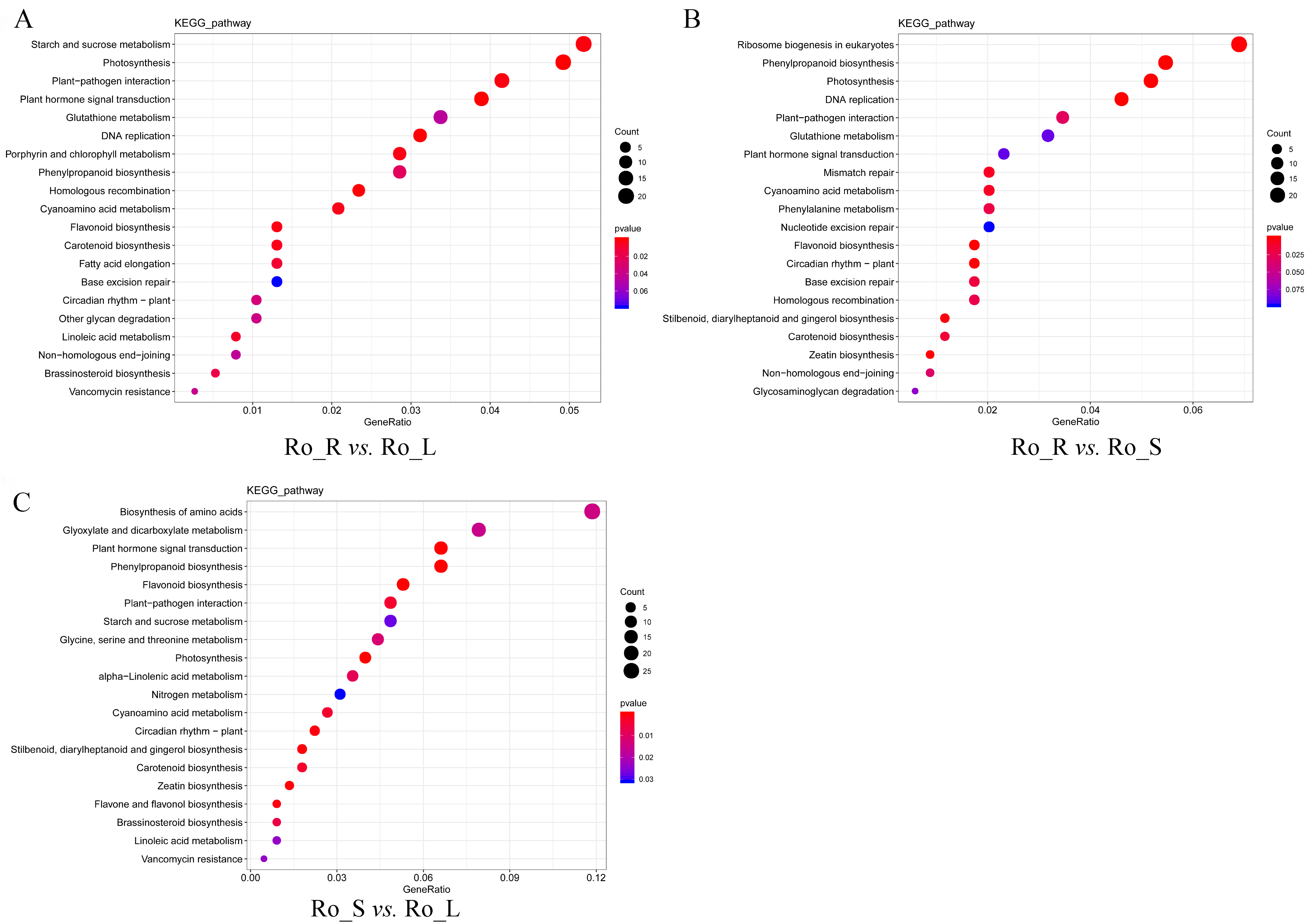

Supplement: Supplementary file 1 [file genes-13-01592-s001.zip › Figure S5.tif]

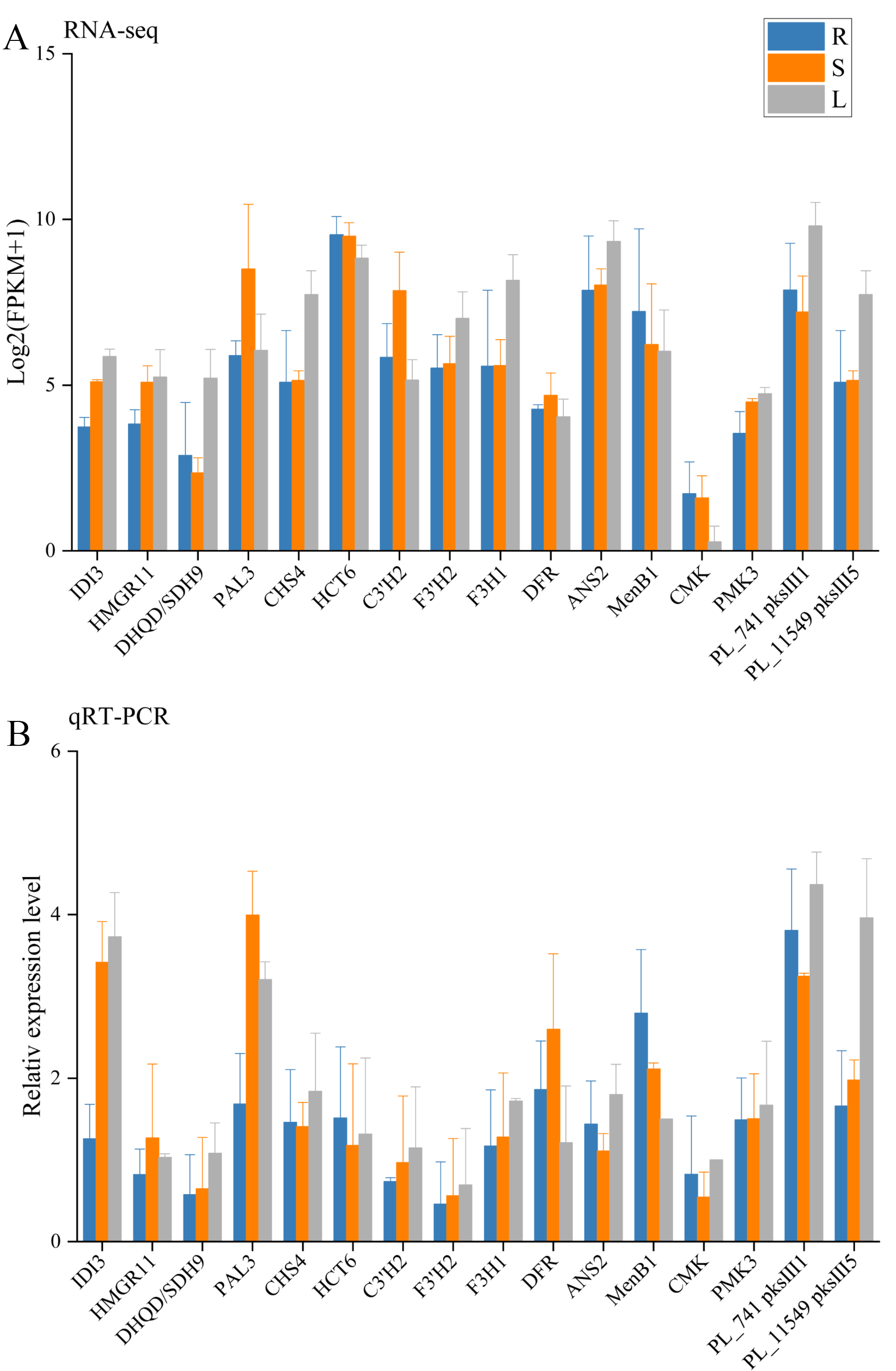

Supplement: Supplementary file 1 [file genes-13-01592-s001.zip › Figure S6.tif]

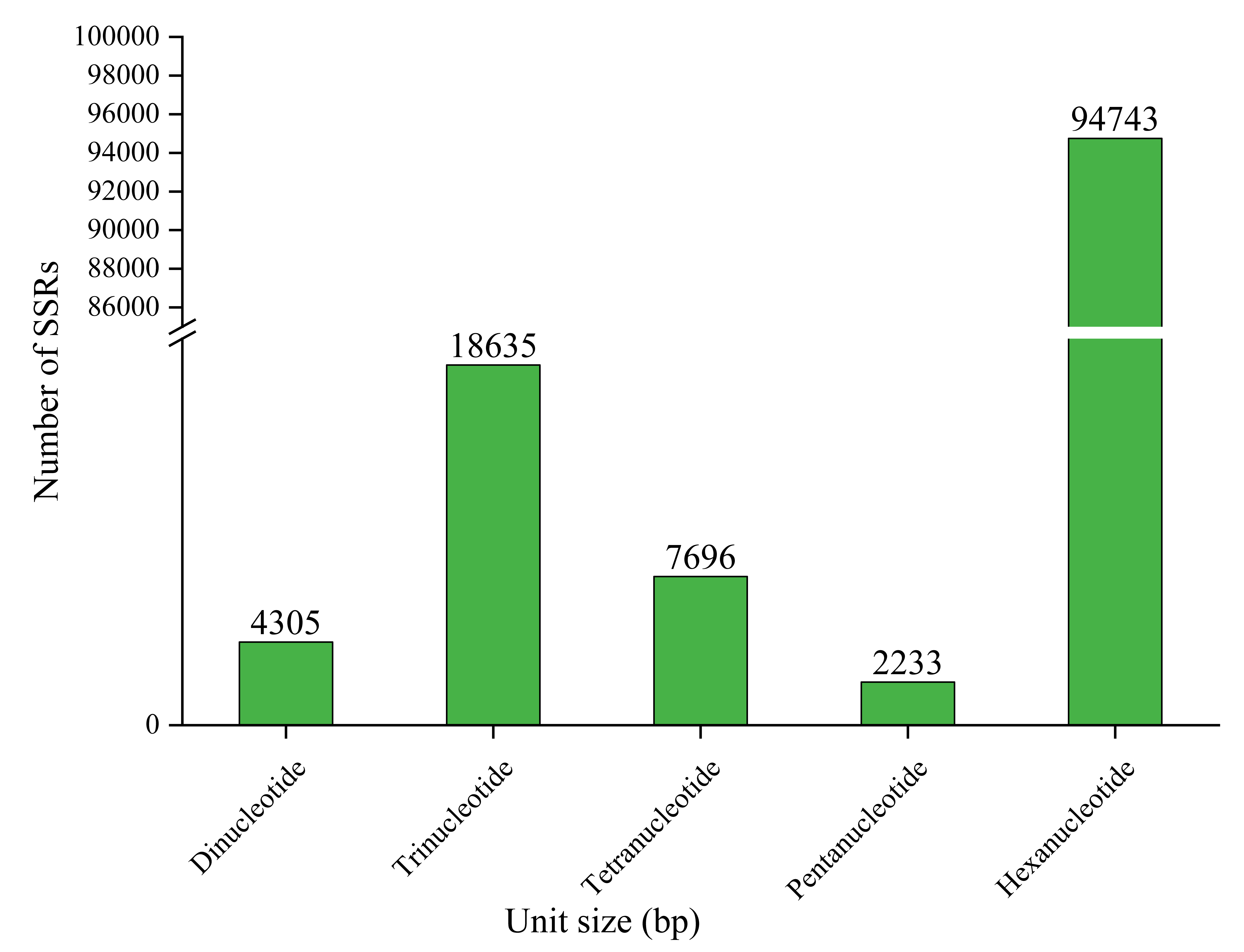

Supplement: Supplementary file 1 [file genes-13-01592-s001.zip › Figure S7.tif]
